# Interspecific Comparison of the Performance of Soaring Migrants in Relation to Morphology, Meteorological Conditions and Migration Strategies

**DOI:** 10.1371/journal.pone.0039833

**Published:** 2012-07-02

**Authors:** Ugo Mellone, Raymond H. G. Klaassen, Clara García-Ripollés, Ruben Limiñana, Pascual López-López, Diego Pavón, Roine Strandberg, Vicente Urios, Michalis Vardakis, Thomas Alerstam

**Affiliations:** 1 Estación Biológica Terra Natura, Vertebrates Zoology Research Group, CIBIO, University of Alicante, Alicante, Spain; 2 Department of Biology, Lund University, Lund, Sweden; 3 Instituto de Investigación en Recursos Cinegéticos (IREC), CSIC-UCLM-JCCM, Ciudad Real, Spain; 4 Bird Ecology Unit, Department of Biosciences, University of Helsinki, Helsinki, Finland; Ohio State University, United States of America

## Abstract

**Background:**

Performance of migrating birds can be affected by a number of intrinsic and extrinsic factors like morphology, meteorological conditions and migration strategies. We compared travel speeds of four raptor species during their crossing of the Sahara desert. Focusing the analyses on this region allows us to compare different species under equivalent conditions in order to disentangle which factors affect migratory performance.

**Methodology/Principal Finding:**

We tracked raptors using GPS satellite transmitters from Sweden, Spain and Italy, and evaluated their migratory performance at both an hourly and a daily scale. Hourly data (flight speed and altitude for intervals of two hours) were analyzed in relation to time of day, species and season, and daily data (distance between roosting sites) in relation to species, season, day length and tailwind support.

**Conclusions/Significance:**

Despite a clear variation in morphology, interspecific differences were generally very small, and did only arise in spring, with long-distance migrants (>5000 km: osprey and Western marsh-harrier) being faster than species that migrate shorter distances (Egyptian vulture and short-toed eagle). Our results suggest that the most important factor explaining hourly variation in flight speed is time of day, while at a daily scale, tailwind support is the most important factor explaining variation in daily distance, raising new questions about the consequences of possible future changes in worldwide wind patterns.

## Introduction

The migratory performance of birds can be shaped by intrinsic factors like species morphology, experience and migration strategy [1]–[3] and also by extrinsic factors like landscape properties and meteorological variables [4]–[7]. Diurnal migrants flying over land can migrate by flapping or soaring flight. As energy consumption during flapping flight increases steeply with body mass, soaring migration is more advantageous for larger bird species [8], [9]. However, soaring flight requires thermal updrafts that develop only over land and only during the day and hence, soaring migrants are more dependent on topography and circadian patterns in thermal convection than birds using flapping flight [10].

Here, we analyze migratory performance of four species of diurnal raptors using GPS satellite telemetry. We analyze a set of species tagged in different regions of Europe, differing greatly in morphology and behaviour, but sharing basically the same flyway and migration period, and thus facing the same environmental conditions during migration. We restricted our analysis to the crossing of the Sahara desert in order to avoid any variation related to food searching behaviour. Since the desert is an ecological barrier [5], [11], all the species probably cross it as efficiently and safely as possible, allowing for comparison under equivalent conditions.

We explore the general performance of soaring migrants crossing the Sahara desert, investigating factors like cross-country speed, flight altitude, daily migration time, daily migration distance, and effects of wind and thermal convection on resulting speed. It is only through the novel high-resolution satellite tracking of migrants that it is now possible to study these basic aspects of the performance of migratory birds at hourly and daily time scales [4]. In this, we compare different species making the same crossing of the Sahara desert, including osprey (*Pandion haliaetus*), Western marsh-harrier (*Circus aeruginosus*), Egyptian vulture (*Neophron percnopterus*) and short-toed eagle (*Circaetus gallicus*). These four species differ with respect to body mass and morphology as well as to migration distances: while ospreys and Western marsh-harriers travel around 5000 km between the breeding and the wintering grounds, Egyptian vultures and short-toed eagles breed roughly 2000 km closer to the wintering grounds (Table 1).

**Table 1:** Sample sizes and characteristics of the four species involved in this study.

Species	N ind	N tracks spring	N tracks autumn	Body mass (kg)	Wing span (m)	Wing area (m^2^)	Aspect ratio	Wing loading (kg/m^2^)	Migration distance (km)
osprey	5	9	13	1.6	1.60	0.32	8.0	4.9	6800
Western marsh-harrier	4	5	7	0.65	1.16	0.20	6.6	3.2	5100
Egyptian vulture	6	9	15	2.1	1.65	0.36	7.7	5.8	3100
short-toed eagle	7	4	11	1.7	1.9	0.41	8.9	4.2	2300–5000

Sources: [8], [27], [34], [36], [41]–[44].

On the basis of these differences, we formulated the following hypothesis and tested the related predictions (Table 2):

**Table 2:** Summary of the hypothesis tested on soaring migrants crossing the Sahara desert.

Hypothesis	Prediction
Wing loading	Species with lower wing fly more hours, while species with higher wing loading show higher gliding speed between thermals
Day length	The day length enhances the daily distance
Spring arrival	Adult individuals migrate faster during spring, due to breeding motivation
Time constraint	Species migrating longer distances (>5000 km) travel faster than those migrating shorter distances

### 1) Wing Loading Hypothesis

Species with lower wing loadings are able to exploit weaker and narrower thermals as they have lower minimum sink speeds and smaller turning radia, and are thus expected to initiate migration earlier in the morning and continue migration until later in the afternoon than species with higher wing loadings [12], [13]. However, the gliding speed between thermals is higher for species with higher wing loadings, which more than compensates for a reduced climbing performance, resulting in higher cross-country speeds [9], [12]. Thus, as soon as the thermal conditions are good enough to support the species with higher wing loadings (i.e. strong and wide thermals), we expect these larger soaring migrants to travel faster than the species with lower wing loadings.

### 2) Day Length Hypothesis

Day length varies according to latitude and date, and might have an effect on migration speed for passerine birds through longer foraging times [14], [15]. Similarly, the time for soaring is longer for longer days, and we thus may expect that day length has a positive effect on the number of travelling hours and hence, on distance covered per day for thermal soaring migrants.

### 3) Spring Arrival Hypothesis

During spring migration the goal of adult individuals is to reach their breeding grounds and start reproduction early and therefore, competition for early arrival may promote a faster migration during spring compared to autumn [16].

### 4) Time Constraint Hypothesis

Species migrating overall longer distances (>5000 km, like the osprey and the Western marsh-harrier; Table 1) are expected to be more time-selected than short-distance migrants [17], [18], and thus should travel faster, achieving higher daily distances.

The two latter hypotheses both suggest that birds use behavioural strategies to increase migration speed and thus reduce the duration of migration, depending on both season (prediction 3) and migration distance (prediction 4) and, as a consequence, the most extreme time-selected strategies are expected for adult individuals of long-distance migrants during spring migration [19].

Moreover, our aim was also to study how meteorological factors like wind conditions and thermal energy interact with the factors discussed in the previous hypothesis, in particular at a daily scale. Wind assistance is of paramount importance, since it can allow birds to increase their flight range irrespectively of fuel expenditure [17], thus saving both time and energy. Thermal convection, the rising movement of a portion of air, is affected by other meteorological factors (temperature gradient with altitude determines atmospheric stability) as well as by topography. Climbing rate is of greatest importance for the resulting cross-country speed of soaring migrants [20] and is dependent upon thermal convection irrespectively of birds’ morphology [8]. Therefore, we investigated the response of birds to these factors (day length, wind, thermal convection) at different temporal scales (hourly and daily).

## Results

**Figure 1:**
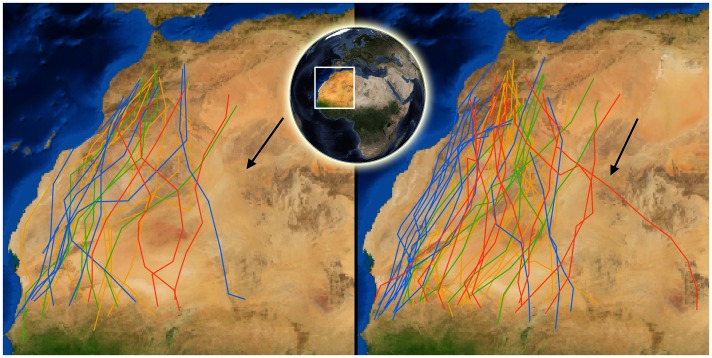
Migration tracks of raptors crossing the Sahara desert. Each segment connects two roosting locations. Spring tracks are on the left and autumn tracks on the right. The colours of the segments indicate the species as Osprey (blue), Marsh Harrier (green), Egyptian Vulture (orange), or Short-toed Eagle (red). Black arrows represent average wind directions (see Results). Sample sizes are given in table 1.

The migratory performance was evaluated for the raptors’ crossing of the Sahara desert along the flight routes shown in Figure 1.

### (a) Hourly Scale

**Figure 2:**
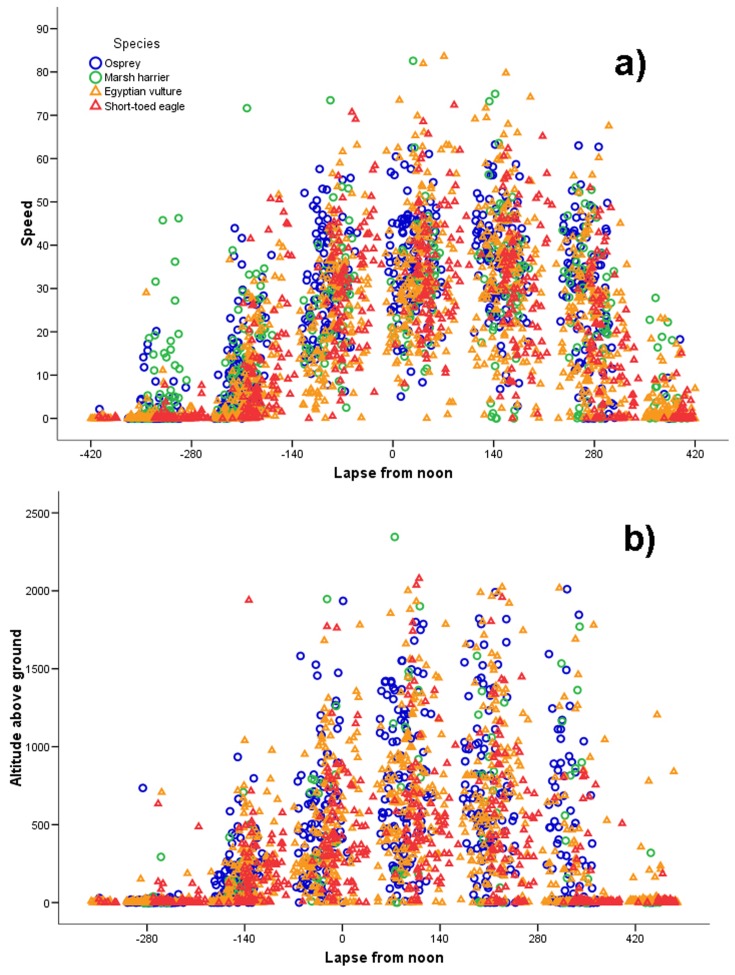
Circadian pattern of speed and altitude. Speed (a; *N* = 2835) is given in km/h and altitude in meters above ground (b; *N* = 2659). Both parameters are plotted in relation to time of day (calculated as minutes from noon, see Methods for details).

**Figure 3:**
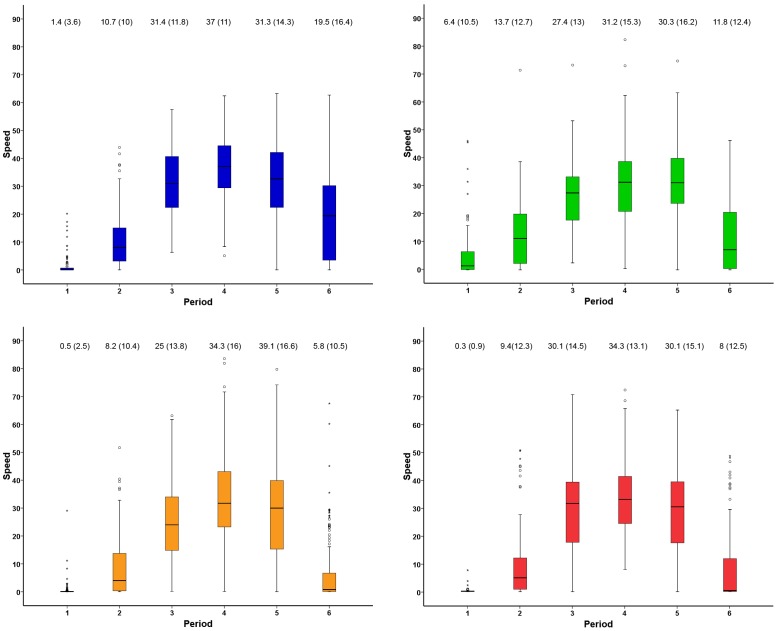
Boxplots showing speed (km/h) in relation to time of day categories (see Methods). The colours indicate the species as Osprey (blue), Marsh Harrier (green), Egyptian Vulture (orange) and Short-toed Eagle (red). For each time category the average speed (SD) is given, including both stopping and travelling segments.

**Figure 4:**
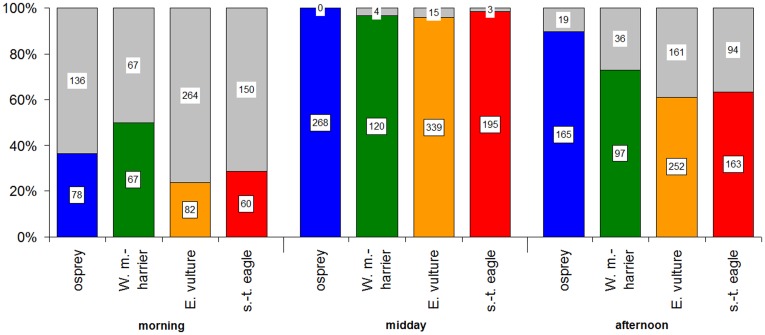
Time budgets and sample sizes in relation to time of day (*N* = 2835). Coloured parts indicate travelling segments, while grey parts represent stopping segments. Morning includes period 1 and 2, midday period 3 and 4, and afternoon 5 and 6 (see Methods).

Concerning the circadian pattern of migration, speeds were generally slower early in the morning and late in the afternoon, being much higher around noon. They showed an asymmetrical distribution in relation to local noon (Figure 2a), with some differences between species. The highest cross-country speeds were attained soon after local noon, when speeds were most commonly in the range 20–60 km/h (Figure 2a). In particular, Western marsh-harriers started movements earlier in the morning than the other species, showing higher speeds during the first of the six periods of the day (Figure 2a and 3; Kruskal-Wallis test; *H*_3, 443_ = 63.78, *p*<0.001; see also figure 4) but flying slower just after midday (period four; Kruskal-Wallis *H*_3, 516_ = 14.01, *p* = 0.003; see average values in Figure 3). The same difference in the fourth period also occurred when taking into account only travelling segments (with movement >5 km/h; cf methods), with the Western marsh-harrier being the slower species (Kruskal-Wallis *H*_3, 507_ = 10.92, *p* = 0.012).

Hourly flight speeds were significantly different among species only during spring (based on travelling segments within all time periods; ANOVA *F*_3,617_ = 7.26, *p*<0.001; autumn: n.s.), with a Tukey post-hoc test revealing that this was due to the difference between ospreys and Egyptian vultures (*p*<0.001).

Analyzing the hourly speed data (travelling segments) separately for each species showed that seasonal differences occurred in the osprey, being faster during spring (speed_spring_  = 33.1 km/h vs. speed_autumn_  = 29.7 km/h; *F*_1,509_ = 7.3, *p* = 0.007), and in the Egyptian vulture, being faster in autumn (26.5_spring_ vs. 29.9_autumn_ km/h; *F*_1,671_ = 7.9, *p* = 0.005).

Altitude values showed a similar pattern as speeds, being asymmetrical with respect to midday, and the highest altitudes, commonly ranging between 200 m and 1500 m above the ground, were recorded during the four afternoon hours immediately after local noon (Figure 2b). Taking into account only altitude data related to travelling segments there was a significant variation among the four species (Kruskal-Wallis *H*_3, 1650_ = 11.34, *p* = 0.01), with short-toed eagles flying on average lower than the other species (Mann-Whitney *U* test, all the three post-hoc comparisons *p*<0.05) while there were no significant altitude differences among flight altitude of ospreys, Western marsh-harriers and Egyptian vultures.

### (b) Daily Scale

Concerning travelling hours, there were interspecific differences during both seasons (Table S1; ANOVA spring: *F*_3,128_ = 18.17, *p*<0.001; autumn: *F*_3,242_ = 6.95, *p*<0.001) with Western marsh-harriers and ospreys spending more time flying than the other two species during both seasons (Tukey test: *p*<0.05).

**Figure 5:**
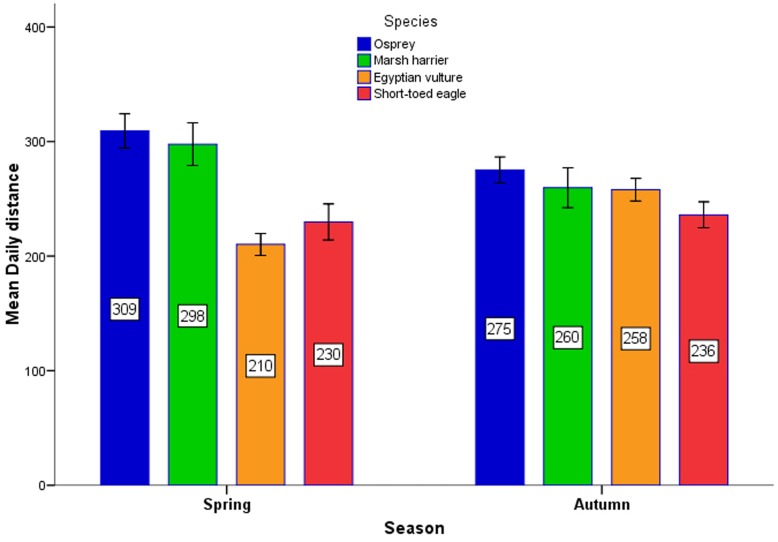
Mean daily distances (±1 SE; N = 547 segments) per season and species. Sample sizes are given in table S2.

The model concerning daily distance and including all data showed a significant effect of “species” and its interaction with “season” (Table 3). In particular, during spring there were significant differences among the four species (ANOVA, *F*_3,196_ = 14.25, *p*<0.001), with the Egyptian vulture and the short-toed eagle travelling shorter daily distances than the osprey and the western marsh-harrier (Tukey test, *p*<0.05; see mean values in Figure 5). In contrast, during autumn, there were no significant interspecific differences (*F*_3,342_ = 1.9, *p*>0.05).

Concerning the species-specific models, in all species a positive effect of “tailwind” arose, and in all species except the short-toed eagle also “season” was significant (Table 4). Thus, for these species, when controlling for other factors, daily distances were significantly higher during spring than during autumn (Table 4). In the Egyptian vulture there were also significant effects of the interactions “season*day length”, and “season*tailwind” (Table 4). When investigating the nature of these interactions it appeared that the effect of day length on daily distance was negative during spring and positive during autumn. However, it should be noted that during spring this effect can be discarded as spurious, since “day length” correlated negatively with “tailwind” (R =  −0.34, *p* = 0.001, N = 89), while during autumn there was no such relationship. Therefore, during spring tailwind had an overriding effect, whereas during autumn daily distance was affected by both following winds and day length. Concerning the interaction “season*tailwind”, the effect of tailwind was positive during both seasons, but stronger during autumn (slopes of regressions of daily distance vs. tailwind: spring  = 9.8 km/day for every meter/sec of tailwind; autumn  = 18.5).

### (c) Spatial and Seasonal Variation of Environmental Factors

**Figure 6:**
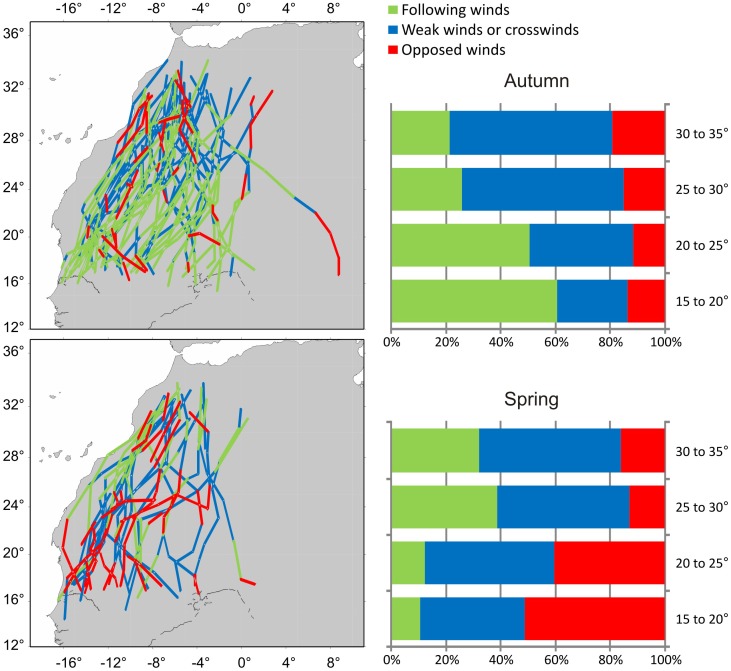
Spatial and temporal variation of wind assistance (all species pooled). The maps show daily segments. Colours indicate the extent of wind support: green  =  tailwind (>2.5 m/s), blue  =  weak wind (2.5 to –2.5 m/s), red  =  headwind (< –2.5 m/s). The bars show the frequency of different tailwind classes in different latitudinal bands.

Average absolute wind conditions (direction and speed) were similar in the two seasons (spring: 36°±91.2, 6.1±3 m/sec; autumn: 26°±86.3, 5±2.7 m/sec; Figure 1). Tailwinds were positively related to latitude during spring and negatively during autumn (spring: R = 0.42, *p* = 0.0001, N = 200; autumn: R =  −0.32, *p* = 0.0001, N = 347; Figure 6), this is to say that during both seasons wind assistance was stronger for the last part of the Sahara crossing. Furthermore, winds were always more favourable during autumn than during spring, in all four species (all four comparisons *p*<0.006; Figure 6).

**Table 3:** GLM mixed model with daily distance as dependent variable, including all data.

Source	Denominator df	F	Sig.
Intercept	15.5	2619.2	.000
Species	14.2	10.2	.001
Season	529.1	.2	.649
Species*Season	519.6	5.3	.001

**Table 4:** Final GLM models for each species, with daily distance as dependent variable.

	Wald	Sig.	Spring	Autumn
*osprey*				
Intercept	1278.5	.000		
Tailwind	15.55	.000		
Season	60.5	.000	328.7 (13)	265 (9.3)
*Western marsh-harrier*				
Intercept	528.9	.000		
Tailwind	16	.000		
Season	27.1	.000	340.6 (20.1)	234.3 (15)
*Egyptian vulture*				
Intercept	1.5	.215		
Tailwind	7.3	.007		
Season	89.5	.000	214.1 (18.5)	208.2 (11.4)
Day length	3.6	.067		
Day length*Season	7.1	.008		
Tailwind*Season	7.8	.005		
*short-toed eagle*				
Intercept	697.9	.000		
Tailwind	32.9	.000		
Season		n.s.	233.7 (8.2)	233.7 (8.2)

For each season, the marginal means of daily distance (km/day; with SD in parentheses), corrected for effects of other significant factors besides season (tailwind, day length), are given). See Methods for details.

**Figure 7:**
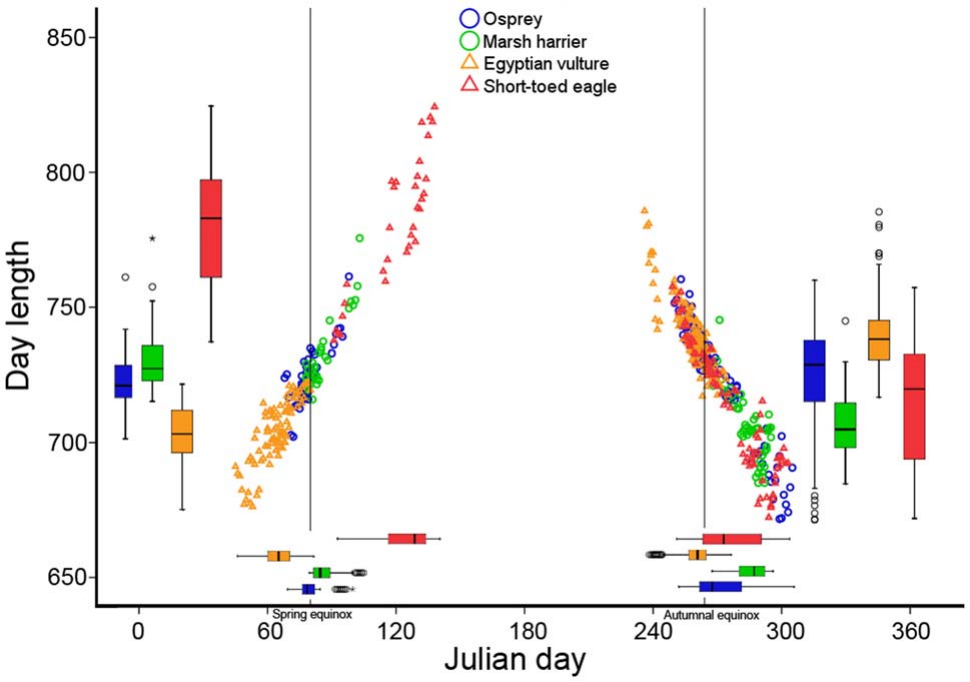
Day length experienced by the birds during each daily segment, plotted against Julian day. The same variables are expressed also as box-plots.

Day length was longer during spring than during autumn for Western marsh-harriers and short-toed eagles, and conversely for the Egyptian vulture (all *p*<0.001; Figure 7), while there was no difference for the osprey. Therefore, the Egyptian vulture was the only species experiencing more favourable factors simultaneously (tailwind and day length) in the same season (autumn).

## Discussion

In this study we compared the behaviour and migratory performance of different bird species migrating across the same regions and during the same periods. Our results showed that migratory performance is remarkably similar across species that differ greatly in morphology and breeding range. Despite these general similarities in performance, we also found that some responses to external factors are season- and species-specific.

### Influence of Morphology

In agreement with our predictions based on morphology, the Western marsh-harrier was the species initiating its daily flight first, showing fast movements early in the morning, when the other species were still mostly at their night roosting sites. However, in agreement again with predictions based on morphology, the harriers travelled at lower speeds than the other larger raptor species after midday, which is the best time for soaring flight (Figure 2a and 3). Thus, the three heavier species seem to take advantage of the stronger thermals that occur during the central hours of the day whereas the Western marsh-harrier exploit also weaker thermal conditions or use flapping flight early in the morning to a higher degree than the other species. By starting migration earlier and thus flying more hours per day (Figure 2a), Western marsh-harriers covered the same daily flight distance as for example the osprey (Figure 5), despite a slower hourly speed at noon when thermals were strongest. Overall, both hourly speed and altitude showed the same diurnal distribution, peaking just after midday (Figure 2), presumably due to a time lag between irradiation by the sun and the development of thermals.

### Influence of Day Length

We found only little support for our second prediction that day length would have a positive effect on daily distance. In the Egyptian vulture, during spring, winds had an overriding effect, and as wind were more favourable in the northern part of the crossing (Figure 6), where days were shorter, the strong positive effect of wind hides any effect of day length. During autumn, there was no such correlation between wind and day length, but both factors seemed to enhance daily distance simultaneously, increasing the flight range of Egyptian vultures. Unexpectedly, for the short-toed eagle, there was no relationship between daily distance and day length in any season, despite this species is a typical soaring migrant [8]. In addition, there was no seasonal difference in daily distance for this species. A explanation for the lack of day length and seasonal effects in this species would be that they were non-breeders, probably less time-constrained and hence not fully motivated to use the whole daily thermal soaring window to reach their destinations as fast as possible. Thus, in the future it would be interesting to analyze the behaviour of adult short-toed eagles, perhaps more prone to take advantage of the whole daily flight window and thus more dependent on day length.

### Influence of Thermal Conditions

Considering the time of the day as a proxy of the strength of thermal currents, we showed that, at an hourly scale, thermal convection had a profound effect on the diurnal variation of speed and altitude (Figure 2). The large variation in flight altitude shows wide scatters also within the same periods (Figure 2b). Raptors constantly change altitude from a few hundred metres above ground, where they start to climb in a new thermal, up to high altitudes, sometimes approaching 2000 m above the ground, where they leave the thermal to glide off in the migratory direction. At the daily scale, we excluded thermal energy from the models (see Methods) and we found a strong effect of winds on all species and seasons. Our findings are of broad interest, but it must be considered that they are related only to the Sahara desert, where thermal conditions are probably generally very good (being thus less likely than wind to be an important agent affecting daily distance) and therefore our analyses fail to demonstrate a clear effect of thermal variation on the daily scale. In this scenario, it is interesting to highlight that Mandel et al. [21], found regional differences between different migratory flyways of Turkey vultures (*Cathartes aura*) in North America, with individuals flying over flat plains being more affected by wind conditions than individuals following mountain chains, which were more thermal constrained. Chevallier et al. [22] reported that wind did not significantly affect the daily distance covered by migrating black storks (*Ciconia nigra*) during trans-Saharan soaring migration, but comparison with our results is difficult due to the differences in statistical approaches.

### Differences in Travel Speed between Species and Seasons

Controlling for wind assistance, that was less favourable during spring, at a daily scale the three species involving adult individuals (i.e. excluding short-toed eagles) were faster during spring (Table 4), in agreement with our third prediction (spring migration strategy promoting higher speeds) [16]. This effect was particularly evident for birds that had to cover longer migration distances, (i.e. ospreys and Western marsh-harriers), in agreement with our fourth prediction (individuals migrating longer distances are more time constrained). Perhaps this pattern is a consequence of a trade-off between speed and total duration of migration in respect to the overall migration distance [18], with species facing a longer distance being more time-selected [17]. How did the osprey and Western marsh-harrier achieve higher speeds in spring, after accounting for the effects of wind and other environmental factors, in comparison with autumn and with the other two species? Our results suggested that, while in the case of the Western marsh-harrier the higher daily distances in spring were obtained mostly because of more time spent flying, for the osprey there was also an effect of higher flight speeds. One way of increasing cross-country speed among soaring migrants is to use a strategy of mixed soaring and flapping flight and using “partially powered” glides, as for example common cranes (*Grus grus*) commonly do [23]. The gain in resulting cross-country speed by this strategy comes at an increased cost of energy consumption per unit distance covered compared to pure soaring migration, as evaluated by Pennycuick et al. [23]. It seems likely that ospreys, and perhaps also Western marsh-harriers, adopt such a strategy in order to reduce time to complete their spring migration (at a cost of increased energy consumption) while Egyptian vultures and short-toed eagles behave more strictly as pure soaring migrants. More detailed studies are needed to confirm and evaluate if and to what degree flapping flight is included in the soaring migratory behaviour among different seasons and among different species during their Sahara crossing.

Models revealed that tailwind was by far the best predictor of daily distance, since all species during both seasons were affected by the tailwinds that they experienced during their journeys. In agreement with Shamoun-Baranes et al. [24] tailwinds were more favourable in autumn compared to spring, but this pattern was only reflected in the daily distances covered by the Egyptian vulture, that during autumn also took advantage from longer days (Figure 7). The fact that ospreys and Western marsh-harriers were faster during spring, despite winds being less favourable, highlights the stronger time pressure during this season, where the birds seem to have adopted a strategy to achieve high travel speeds in order to reach the breeding grounds as soon as possible (prediction 3) [16].

Overall, differences among the four species were smaller than expected on the basis of their behaviour when crossing other regions, like the Mediterranean Sea, where the species showed more differences concerning their tendency to cross open water and consequently also in their flight behaviour [25]–[27]. All the species, and especially the three ones with higher wing loading behaved as true soaring migrants, restricting their flight activity to the daily hours when thermals developed. This behaviour is in contrast to species, which in addition to soaring flight, regularly use sustained flapping flight during their Sahara crossing such as the Eurasian hobby (*Falco subbuteo*), the Eleonora’s falcon (*Falco eleonorae*) and the lesser kestrel (*Falco naumanni*) that extend their travelling time also during the night to gain considerably longer daily flight range (sometimes >500 km/day) [5], [28]–[29]. Interspecific differences arose mainly in spring, when osprey and Western marsh-harriers were faster than Egyptian vultures and short-toed eagles, perhaps because of a combined effect of longer total migration distance and breeding pressure to migrate faster (prediction 3 and 4). Future studies including individuals of different species from the same breeding areas and belonging to the same age class could improve our knowledge concerning which factors determine migratory performance of long-distance migrating birds, possibly also using new devices which would allow studying movements at a finer temporal scale than the one presented here. A detailed understanding of the behaviour of migrating birds when crossing the Sahara desert region could help to disentangle the adaptations developed to cope with scarcity of food and water and the harsh weather conditions [30]. This is particularly important also in the light of the deleterious effects on fitness that these birds can experience thousands of kilometres away, at their breeding grounds, in case of adverse conditions met during the crossing of this ecological barrier [11]. In this scenario, it should be noted that, even if there is no clear indication of desert expansion [31], sand storms are becoming much more frequent [32].

In conclusion, our results suggested that, while thermal strength was the most important factor on a hourly basis, at the daily scale wind was more important, allowing birds to significantly increase their daily flight ranges over the Sahara desert. The effect of day length was much less marked if it had any effect at all. The impact of winds upon migration speed raises interesting questions about future effects of global change on migratory perfomance, at least in the light of the predictions concerning worldwide wind patterns [33].

## Materials and Methods

### (a) Selection of Migration Data

We used data from four raptor species tagged with 22 g, 30 g or 45 g ARGOS/GPS PTT-100 from Microwave Telemetry Inc. (horizontal accuracy of locations: ±18 m), in Spain, Italy and Sweden between 2006 and 2010. The dataset included 22 individuals completing 73 journeys (Table 1, Figure 1). Egyptian vultures were tagged as adults in Spain [34], and short-toed eagles as nestlings in Spain and Italy [35], [27]. Ospreys and Western marsh-harriers were tagged as adults in Sweden [4], [36]. Hence, all the tagged individuals apart from the short-toed eagles were experienced breeding adults. As there were no differences in the results of the analyses for short-toed eagles during their first autumn migration (age: juvenile) and the subsequent ones (age: immature), we pooled all the autumn migration data of this species for further analyses. To select the data, we considered as Sahara desert the latitudinal band between 31° and 17.5° N, a journey of ca. 1500 – 2000 km. We included the data between the last roosting site before entering the desert and the first one after the desert crossing. Three sampling units were identified: (i) hourly flight speed; (ii) altitude above ground (accounting for an hourly scale); and (iii) daily distance (accounting for a daily scale) [37]. The hourly flight speed was calculated at two-hour intervals, while the daily distance was calculated as the loxodromic distance between two consecutive roosting sites. A few days with daily distance <50 km were excluded from the analyses [38]. The altitude above ground was obtained for the same two-hour intervals that were used to calculate hourly flight speed, by subtracting the ground altitude (obtained from http://www.geonames.org/) from the bird’s altitude recorded by the transmitter (nominal accuracy ±22 m) at the beginning of the segment.

### (b) Time and Day Length

In order to standardize the time of two-hour segments irrespectively of longitudinal and seasonal differences we calculated the lapse from local noon considering the median time (for speeds) or the ending time (for altitudes). These values were thus negative before local noon and positive afterwards. The time of local noon for each location was calculated according to the formulae provided by NOAA (http://www.ecy.wa.gov/programs/eap/models.html). These time values were grouped into six time categories according to a step of 140 minutes, from early morning (–420,–280), until late afternoon (281, 420), through the intermediate categories (–420,–280;–279,–140; –139,0; 1, 140; 141, 280; 281, 420).

Day length was expressed as that one experienced by each individual throughout a given day, thus as the difference (in minutes) between the sunset at the roosting site of arrival and the sunrise at the roosting site of departure. The exact times of sunrise and sunset for each roosting site (when sun is 0.833 degrees below horizon) were calculated using the abovementioned formulae.

### (c) Meteorological Variables

Data were obtained from the NCEP/NCAR Reanalysis project, as provided by the NOAA/OAR/ESRL PSD, Boulder, CO, USA (http://www.cdc.noaa.gov), using R package RNCEP [39]. Since only four measurements per day were available, we used meteorological variables only for the analyses at the daily scale, collecting the data at 12∶00 hours for the midpoint of each daily segment. Wind data (U and V wind components) were extracted for a pressure level of 925 hPa, which corresponds to an altitude of about 750 m a.s.l, and thus was the closer measurement to the cruising altitude of our tracked birds [38]. The U and V wind components were combined into single wind vectors from which we calculated a tailwind component in relation to the overall direction of each journey as the wind speed multiplied by cosine for the angle between wind and migration direction. Conditions characterized by tailwind component >2.5 m/s were designated as “following winds”, tailwind component between 2.5 and –2.5 m/s as “weak winds or crosswinds” and tailwind component < –2.5 m/s as “opposed winds” (Figure 6).

Concerning thermal energy, we performed exploratory analyses testing the effect of two different variables: 1) a temperature gradient (°C/100 m) following Chevallier et al [22], and 2) the velocity of thermal convection (w*), following Bohrer et al. [7]. In both cases, results were non-significant or driven by spurious patterns (e.g., negative relationship of w* with tailwind) leading to a negative effect of thermal conditions on daily speed. We do not present here those results for reasons of space constraints and we run the final models on daily distance excluding any thermal variable.

### (d) Statistical Analyses

After an inspection of the frequency distribution of flight speeds at the two-hour intervals, we divided the segments either as “stopping” or “travelling” according to a threshold of 5 km/h [5], [28]–[29], [40]. Travelling hours were computed as the elapsed time between the onset-time of the first travelling segment of the day and the end-time of the last one, and only for days when the transmitter was working continuosly (thus the sample size was lower), in order to detect accurately the beginning and the end of the daily flight. To identify which factors shaped the daily distance, we first ran a General Linear Mixed Model including all data, with “season”, “species” and their interaction as fixed factors, and the individual as random factor. Finally, we ran four separate General Linear Models, one for each species, including, besides “season”, also “tailwind”, and “day length”, as well as the interaction of each one of these terms with “season” (a total of five terms). Non-significant terms were removed stepwise from the models according to their p-value within the model, starting from the interactions, until we obtained models that retained only significant variables. However, if a factor was significant only within an interaction, it was not removed as a single term. Since exploratory analyses showed that, at least in some cases (Table S2), there was an effect of latitude on daily distance, and that latitude was also correlated with wind assistance (see Results), we excluded latitude from the analyses in order to avoid spurious results. The analyses were carried out using SPSS 15.0.

## Supporting Information

Table S1 **Daily travelling hours (sample size, average and standard deviation) for each species by season.**
(DOC)

Table S2 **Regression coefficient and significance of the relationship between daily distance and latitude for each species by season.**
(DOC)
