# Supplementary material for: Interspecific Comparison of the Performance of Soaring Migrants in Relation to Morphology, Meteorological Conditions and Migration Strategies
Source: PLoS One. 2012 Jul 2;7(7):e39833. doi: 10.1371/journal.pone.0039833 (PMC3388085; doi:10.1371/journal.pone.0039833)
Supplement: Table S1 — Daily travelling hours (sample size, average and standard deviation) for each species by season. (DOC) [file pone.0039833.s001.doc]

**Table S1. Daily travelling hours (sample size, average and standard deviation) for each species by season.**

|  | Spring | | | Autumn | | |
| --- | --- | --- | --- | --- | --- | --- |
|  | N | Mean | S.D. | N | Mean | S.D. |
| osprey | 35 | 9.8 | 1.3 | 72 | 9.6 | 1.8 |
| Western marsh-harrier | 27 | 10.3 | 1.7 | 41 | 9.9 | 2.5 |
| Egyptian vulture | 49 | 8.2 | 1.5 | 72 | 8.7 | 1.9 |
| short-toed eagle | 21 | 8.1 | 1.2 | 61 | 8.6 | 1.6 |
