# Supplementary material for: Interspecific Comparison of the Performance of Soaring Migrants in Relation to Morphology, Meteorological Conditions and Migration Strategies
Source: PLoS One. 2012 Jul 2;7(7):e39833. doi: 10.1371/journal.pone.0039833 (PMC3388085; doi:10.1371/journal.pone.0039833)
Supplement: Table S2 — Regression coefficient and significance of the relationship between daily distance and latitude for each species by season. (DOC) [file pone.0039833.s002.doc]

**Table S2. Regression coefficient and significance of the relationship between daily distance and latitude for each species by season.**

|  | Spring | | | Autumn | | |
| --- | --- | --- | --- | --- | --- | --- |
|  | N | R | P | N | R | P |
| osprey | 47 | **0.56** | **0.001** | 89 | -0.18 | 0.098 |
| Western marsh-harrier | 32 | **0.43** | **0.014** | 54 | -0.12 | 0.393 |
| Egyptian vulture | 89 | 0.20 | 0.066 | 116 | 0.10 | 0.300 |
| short-toed eagle | 32 | 0.17 | 0.355 | 89 | -0.17 | 0.112 |
